# Spatial Analysis of Healthcare Offer and Request for Older People Aged 65 Years and Over in Quebec

**DOI:** 10.1093/geroni/igaa057.3327

**Published:** 2020-12-16

**Authors:** Juliette Duc, Sébastien Barbat-Artigas, Delphine Bosson-Rieutort

**Affiliations:** 1 School of Public Health, University of Montréal, Amiens, France; 2 INESSS, Montréal, Canada

## Abstract

With years, the health-needs of an individual become numerous and more complex, resulting in the requirement of an even more appropriate offer of health services. However, it is known that different factors make the services and the request of the population to health care unequally, especially the interregional variations. These are represented by a gap between the offer of healthcare and the need of the populations. The aim of this study was 1) to map the relationship between the location of the healthcare services in Quebec and population aged 65 years and over, and 2) to identify the characteristics related to the geographic variations in access to healthcare. We used data from “statcan.gc.ca”, “donneesquebec.ca” and “msss.gouv.qc.ca” regarding the facilities, their capacity, their services, and the populations’ characteristics. Analyses were performed on QGIS and R software. As expected, our results showed that there is a gap between the healthcare needs and the services: older people need a large amount of diverse services which are not always provided by secluded areas. Moreover, it also appeared that the deprivation index is related to the offer of health care. As this project takes part in a global project studying the health care trajectories of older people in Quebec using their administrative health databases, those findings will help better understand the impact of the geographic factors for the interregional variations of healthcare.

